# AAV Engineering for Improving Tropism to the Central Nervous System

**DOI:** 10.3390/biology12020186

**Published:** 2023-01-26

**Authors:** Muhammad S. Ghauri, Li Ou

**Affiliations:** 1 School of Medicine, California University of Science and Medicine, Colton, CA 92324, USA; 2 Genemagic Biosciences, Media, PA 19086, USA; 3 Department of Pediatrics, University of Minnesota, Minneapolis, MN 55454, USA

**Keywords:** AAV, capsid engineering, CNS, blood-brain-barrier, directed evolution, in silico

## Abstract

**Simple Summary:**

Adeno-associated virus (AAV) is a small, non-pathogenic, and replication-defective virus that mainly infects primates. AAV has demonstrated great success in pre-clinical and clinical applications, including central nervous system (CNS), ocular, muscular, and liver diseases. We have encountered a variety of obstacles, such as delivery efficiency, packaging optimization, and immunogenicity that have hindered the therapeutic potential of AAV gene delivery. Much progress has been made to enhance AAV trophism to the CNS while de-targeting peripheral organs such as the liver, to minimize toxicity. However, the blood-brain barrier (BBB), remains a significant challenge for clinical applications, complicating vector delivery into and within various CNS compartments. Here, we outline the key studies utilizing AAV engineering methods including directed evolution, rational design, and in silico design that have been developed to accelerate the discovery and translation of novel CNS capsids.

**Abstract:**

Adeno-associated virus (AAV) is a non-pathogenic virus that mainly infects primates with the help of adenoviruses. AAV is being widely used as a delivery vector for in vivo gene therapy, as evidenced by five currently approved drugs and more than 255 clinical trials across the world. Due to its relatively low immunogenicity and toxicity, sustained efficacy, and broad tropism, AAV holds great promise for treating many indications, including central nervous system (CNS), ocular, muscular, and liver diseases. However, low delivery efficiency, especially for the CNS due to the blood-brain barrier (BBB), remains a significant challenge for more clinical application of AAV gene therapy. Thus, there is an urgent need for utilizing AAV engineering to discover next-generation capsids with improved properties, e.g., enhanced BBB penetrance, lower immunogenicity, and higher packaging efficiency. AAV engineering methods, including directed evolution, rational design, and in silico design, have been developed, resulting in the discovery of novel capsids (e.g., PhP.B, B10, PAL1A/B/C). In this review, we discuss key studies that identified engineered CNS capsids and/or established methodological improvements. Further, we also discussed important issues that need to be addressed, including cross-species translatability, cell specificity, and modular engineering to improve multiple properties simultaneously.

## 1. Introduction

**Figure 1:**
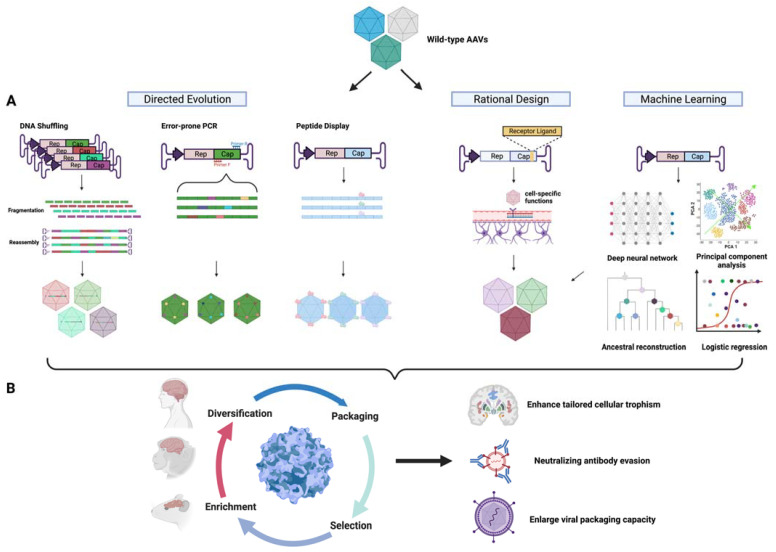
Schematic diagram of capsid engineering to improve CNS trophism. (**A**) Methods for AAV capsid diversification. (**B**) Workflow for capsid engineering, including multi-species selection and tailored enhancement of cellular trophism, immune evasion, and viral packaging.

Adenovirus-associated virus (AAV) is a small, non-pathogenic, and replication-defective virus that infects some primate species, including humans [1]. AAV is composed of protein capsids and linear single-stranded DNA, encoding genes essential for its life cycle. Recombinant AAV, which uses a vector to deliver therapeutic transgenes, rarely integrates into the host cell genome and mainly functions as an episome [2]. However, a recent canine study showed AAV integration events and clonal expansion [3]. This study highlights the need for long-term monitoring for potential genotoxicity [4,5]. Currently, AAV is the mainstream delivery method for in vivo gene therapy. Until June 1, 2022, AAV-mediated gene therapy has been or is being evaluated in a total of 255 clinical trials [6]. The indications span from the central nervous system (CNS), ocular, muscular, and metabolic diseases to aging, AIDS, and cocaine use. The first-ever approved AAV gene therapy was Glybera for treating lipoprotein lipase deficiency; however, it was withdrawn from the market in 2017. Since then, another five AAV gene therapies have been approved in the United States and/or European Union (EU), three in 2022 alone (summarized in Table 1). These clinical trials and approvals have largely demonstrated the sustained efficacy, relatively low immunogenicity, and toxicity of AAV in general. Recently, incidences of severe toxicity, even patient deaths [7], have raised safety concerns about AAV gene therapy. Multiple methods have been developed to limit or minimize the impact of immune responses against AAV capsids or transgene products. These efforts include the usage of immunosuppressants, capsid decoys, plasmapheresis, IgG protease, CpG depletion, and the induction of regulatory T cells [8]. However, immune responses still represent a major challenge for AAV gene therapy, limiting patient eligibility, increasing safety risk, and reducing efficacy durability. Furthermore, the delivery efficiency of current AAV vectors also needs to be improved significantly. For instance, due to the blood-brain barrier (BBB), delivery to the CNS remains difficult. Despite direct injections into the brain that have been attempted in multiple preclinical and clinical studies [9,10,11], it is still challenging to achieve efficient and uniform biodistribution [12]. In addition, delivery to certain organs, e.g., kidney, heart, pancreas, or specific cell populations, e.g., neurons and microglia, is even more challenging. Therefore, novel AAV capsids with improved properties are highly demanded, as evidenced by multiple recent licensing deals on next-generation AAV vectors. One of the hottest areas is novel capsids for CNS delivery because CNS diseases like Parkinson’s and Alzheimer’s disease represent a huge burden to the healthcare system, especially when taking the aging population into consideration. There have been many endeavors in AAV capsid engineering to discover novel capsids for improved CNS delivery. As illustrated in Figure 1, these methods can be categorized into rational design, directed evolution, and in silico design. In this review, we summarize important studies and milestones in each category and highlight future directions and issues that need to be addressed in this field. In addition, the advantages and disadvantages of each method are summarized in Table 2.

## 2. Capsid Engineering Techniques for Improved CNS Tropism

### 2.1. Rational Design

Rational design is a method of capsid engineering where a priori knowledge of AAV biology is harnessed to diversify capsid variants [13]. These capsids are subjected to an iterative evaluation and refinement to enhance specific functions such as receptor binding, entry, and/or intracellular trafficking [13,14,15,16,17,18]. To guide rational design, a comprehensive understanding of AAV biology is required to identify the possible avenues for capsid engineering [13,14]. Mutational analysis was an early attempt to scan the AAV genome looking for the key players influencing capsid assembly, packaging, and infectivity [13,19]. This led to important discoveries such as the heparin binding site and VP1-3 capsid proteins, as well as important surface-exposed residues that can modulate biodistribution [13,14,15,16,17,18,19,20,21]. Some of the earliest breakthroughs in rational design harnessed site-directed mutagenesis of surface-exposed tyrosine residues, leading to markedly improved CNS transduction. These efforts were initially serotype specific, leading to enhanced AAV2 neuronal transduction in hippocampal and striatal regions [13,14,21]. After an improved understanding of ancestral cap sequences, the disruption of native cellular binding motifs led to improved CNS transduction with AAV6 [22], AAV8 [23,24,25,26], and AAV9 serotypes [27,28]. Similar mutagenesis techniques were employed on surface residues to evade the ubiquitin–proteasome pathway, a potent obstacle to proper AAV intracellular trafficking [28]. These engineered capsids led to a significant reduction in virion ubiquitination, mitigating target recognition for proteasomal degradation, and prolonging efficacy durability [21]. This approach accelerated the infective capabilities of AAV vectors by increasing efficient second-strand synthesis and subsequent nuclear transport [15,17,22,25].

In addition to mutagenesis, the insertion of high-affinity ligands/peptides within the Cap sequence further expanded the transducing capabilities of AAV vectors [24,27,28]. Since wild-type AAVs have natural tropism for multiple target organs, systemic gene therapy becomes complicated due to the possibility of off-target tissue transduction and integration [29]. Rational design has been employed to insert cell-targeting peptides within permissible sites of the AAV genome (e.g., residues 587-588 of the VP1 gene), identified via bacteriophage display libraries [24,25,26,27,28]. This approach can reduce a vector’s natural tropism to peripheral organ targets and divert tropism to recognize and bind to specific CNS tissue receptors that are accessible through the blood [28,29,30,31]. However, one of the largest roadblocks to accessing specific CNS compartments is crossing the blood-brain barrier (BBB) [32]. To circumvent these issues, rational design was used to incorporate BBB shuttle peptides (e.g., THR) that interact with AAV to increase BBB transcytosis and subsequent CNS transduction [24,25,26,27]. This expanded the therapeutic potential of delivering viral vectors to potentially correct neurological diseases [32].

Although highly engineered capsids with increased transduction rates can increase the therapeutic capabilities of viral vectors by lowering therapeutically relevant doses while increasing payload delivery, studies have shown the increased risk of immunogenicity following the transduction of human cells [32,33]. This required engineering efforts that use rational design to strategically evade or dampen the innate immune response [17]. Rosario et al. utilized mutated surface tyrosine residues and microglia-specific promoters (F4/80 or CD68) to transduce microglial compartments, thereby reducing astrogliosis and modulating the immune microenvironment within the CNS [22]. Overall, capsid engineering by rational design led to enhancements in transduction, intracellular trafficking, and immune escape [32]. These advancements allowed for significant strides in CNS gene therapy, as seen with lysosomal storage diseases like mucopolysaccharidosis [25,34], glioblastoma multiforme [27,28,35], spinal muscular atrophy [36,37], amyotrophic lateral sclerosis [28,29,30,31,32,33,34,35,36,37,38,39,40], or Huntington disease [31,32,33,34,35,36,37,38,39,40,41,42,43].

### 2.2. Directed Evolution

Due to the simplistic organization of the AAV viral genome, we have refined our knowledge of the wild-type AAV capsid structure, which naturally assembles with low mosaicism and high genome-capsid correlation [44,45]. These properties can be harnessed to undergo directed evolution, which employs random iterative processes (e.g., DNA shuffling, error-prone PCR, and peptide display) to subject capsid variants to cycles of diversification coupled with selective pressures to identify capsids with improved receptor binding, neutralizing antibody-evasion, and enhanced cellular tropism [45,46]. Unlike rational design, the coupling of random diversification and highly tailored selection enables the generation of significantly improved functionality, even if the mechanism of action is unknown [47,48].

Directed evolution was first introduced to alter the cellular trophism of native AAV capsids. The most simplistic modality of generating diverse libraries involves error-prone PCR, which introduces random point mutations into the AAV cap sequence [46,47,48,49]. Error-prone PCR has demonstrated success within the AAV2 cap gene, unlocking retrograde access to various corticopontine circuits and projection neurons [50]. This approach has been recapitulated to introduce mutations into multiple AAV serotypes for subsequent selection. Error-prone PCR generates minute changes within capsids, but when combined with other diversification strategies, such as DNA shuffling and random peptide display, it can further optimize capsid variants to expand the therapeutic capabilities of gene delivery.

DNA shuffling involves the fragmentation and recombination of capsid DNA sequences to generate diverse libraries. Mahershni et al. applied this technique, combined with error-prone PCR, to the AAV2 capsid gene to improve the functional escape of neutralizing antibodies [49]. Similar applications were conducted for other serotypes (e.g., AAV2, 8, and 9) to develop novel capsids that enhance transduction efficiencies in peripheral tissues such as muscle cells [51,52], adipose tissue [53], lung alveoli, and retinal endothelial [48]. Progress has been made to develop capsids (e.g., AAV-B1) that effectively penetrate the blood-brain barrier (BBB) [54] and various compartments of the central nervous system, including multiple regions of the cerebral cortex [51], the hippocampus [54], astrocytes [53,55], microglial cells [56], glioma cells [57], and spinal cord cells [58].

Girod et al. pioneered the early successes of peptide display within the context of AAV capsid engineering [59]. Peptide display is a flexible technique in which brief segments of amino acid stretches are inserted into exposed AAV capsid areas, thereby increasing viral particle surface presentation to alter cellular trophism. Most CNS-targeting capsids were discovered through 7-mer randomized peptide insertions between 588–589 surface loops [60,61], as seen in capsids such as PhP.B [62], PhP.eB [62,63,64], BR1 [65], MACPNS1/2 [66]. Similar efforts led to multiplex engineering to concomitantly engineer other surface moieties (e.g., 452), leading to the discovery of AAV.CAP.B10 that significantly detargeted the liver and other peripheral organs [66,67]. Others combined multiple techniques of directed evolution to generate libraries that successfully transduced multiple species (rat, mouse, primate, and human) with specificity to neural stem cells [46,68] and brain endothelial cells [69].

Despite marked success in overall CNS transduction, several platforms were created to overcome the cellular heterogeneity responsible for disparate transduction efficiencies within the CNS. The CREATE platform was developed to selectively recover capsids that mediate efficient intracellular trafficking and conversion of single-stranded viral genomes to persistent double-stranded DNA necessary for long-term transduction [62]. Davidsson et al. created the barcoded rational AAV vector evolution (BRAVE), enabling large-scale selection of engineered capsids in a single screening round [63]. Efficient recovery of barcodes from the virally expressed mRNA guaranteed the screening of capsid variants that have successfully undergone all critical steps of AAV infectivity [63]. CREATE was used to further evolve previously discovered capsids such as AAV.PhP.B and AAV.CAP.B10 to further enhance widespread neuronal transduction while lowering the required viral load [64,70]. This platform helped discover the LY6A receptor responsible for AAV binding and transduction, elucidating previously reported species- and strain-specific tropism characteristics [71]. Recent applications of these platforms demonstrated impressive capabilities as a screening tool in rodents before direct screening or validation in non-human primates (NHPs), greatly increasing the cost-effectiveness and throughput of the capsid engineering process. [63,66,67,72,73].

The impact of directed evolution and AAV engineering to abrogate native tropisms and modulate tissue specificity has expanded pre-clinical research applications and unlocked new therapeutic avenues for understanding neurophysiology, disease progression, and gene modulation in the CNS.

### 2.3. In Silico Design

Researchers have also turned to artificial intelligence and in silico design for the discovery of novel capsids with improved features. One study used an ancestral reconstruction algorithm to predict the sequence of putative primordial AAV [74], which is expected to have superior transduction and production profiles [75]. A pool of 2048 capsids was generated, screened, and analyzed individually, resulting in the discovery of Anc80L65. This novel capsid has improved thermostability and comparable production yields over AAV2. Moreover, Anc80L65 was shown to have improved tropism for muscle [74], liver [74], retina [76], kidney mesenchymal cells [77], inner hair cells [78,79], and the CNS [80] in animal models including NHP. Notably, a similar study using the same approach but a different ancestral reconstruction algorithm identified a novel capsid with improved muscle tropism over AAV1 [81]. It was believed that the choice of algorithm and input parameters were equally important to the sequence dataset and that novel computational methods, e.g., machine learning, would be helpful for the search for improved capsids [82]. Recently, the maximum-likelihood ancestral sequence reconstruction (ML-ASR) algorithm was used to identify an amino acid motif within the AAV capsid that is required for liver transduction. Mutating this single G-to-A residue change at position 266 abrogated liver trophism while maintaining trophism in other tissues [83]. This highlights the possibility of engineering capsids that target the liver with minimal amount of sequence changes, thus maintaining the quaternary structure and complex intermolecular interactions needed for capsid assembly [83].

Machine learning, a domain of artificial intelligence, is being widely applied in the fields of computer vision, speech recognition, and medicine. By definition, machine learning refers to the process of improving a set of tasks by building models/algorithms based on training data. In the context of capsid engineering, the first step is to generate an informative training dataset that contains a significant number of ‘retained’ capsids (high-fitness capsids) as positive data and ‘rejected’ capsids (low-fitness capsids) as negative controls. The pool of wildtype AAV capsids, generated through evolution over millions of years, represents a solid positive training dataset that should be used in conjunction with negative datasets, usually obtained through mutagenesis experiments [83]. The pool of ‘retained’ capsids can also be generated in silico through an additive model [84] or stepwise assembly [85]. Then, the second step is to decide on the probabilistic machine learning model, including logistic regression, neural network [86], principal component analysis [84], and autoencoders [83]. It seems that all models were able to identify viable capsids that are distinct from wild-type capsids, but neural networks had more success in deep diversification than logistic regression [82].

Currently, there are no capsids with improved tropism for the CNS generated by machine learning. Most efforts in machine learning-assisted capsid engineering still focus on improving the abilities of packaging [83,84,85,86,87] and immune evasion [86,87,88]. The initial efforts resulted in the identification of a fitness landscape describing specific positions out of 753 amino acids that affect AAV2 packaging as well as a key AAV protein, MAAP [84]. A later study from the same group complemented the earlier study by leveraging neural networks and focusing on a specific region of the AAV capsid protein [86]. Moreover, the effort to improve packaging ability itself is expected to accelerate the process of identifying CNS-tropic capsids by directed evolution. As mentioned earlier, a typical approach for identifying CNS-tropic capsids by directed evolution is random 7 mer insertion at amino acid 588 of the AAV9 capsid protein. While the theoretical diversity is 1.3 billion, a typical experimental library only contains 10^7^ variants due to the limits of molecular cloning experiments. Many variants of this experimental library could not be properly packaged. Thus, to increase the odds of identifying candidate capsids, it is imperative to increase the number of viable capsids in a given library. As shown in a recent study [87], machine learning has successfully optimized the library design, resulting in the identification of capsids that effectively transduce primary human brain tissues.

## 3. Discussion

AAV vectors are the mainstream in vivo delivery method, as evidenced by multiple approvals and over 255 clinical trials over the past 25 years. The main advantages of AAV as a gene therapy vector include: low immunogenicity (compared with adenovirus), low risk of insertional mutagenesis (compared with retroviral and lentiviral vectors), and broad tissue tropism (compared with mainly liver-tropic lipid nanoparticles). However, next-generation vectors are essential as the low delivery efficiency and immunogenicity-related adverse events significantly limit the widest usage of AAV gene therapy. In the past few years, significant progress in AAV engineering has been made, resulting in the discovery and identification of many novel CNS-tropic capsids. There are already multiple clinical trials using engineered AAV capsids (Table 3). In addition, the rapid development of related technologies, e.g., single-cell RNA-seq [89,90,91,92] and machine learning-guided library design [87], has laid a solid foundation for future exciting discoveries in this field. Yet, there are still several issues that need to be carefully considered, as described below.

### 3.1. Cross-Species Translatability

One common observation is that the capsid selected in one species or one strain of a species does not necessarily perform well in another species or another strain of the same species. For instance, PhP.B that showed significant BBB penetrance in the C57 mouse strain [62] could not repeat the success in the BALB/C mouse strain, rats, and NHPs [70,71]. In addition, as shown in a more recent study [73], only capsids selected in cynomolgus macaques, but not those identified in rodents or other NHP strains, could mediate efficient CNS delivery in cynomolgus macaques. Similarly, all macaque-derived capsids were shown to be nonfunctional for BBB penetrance in mice. Indeed, the cross-species translatability of novel capsids represents one of the biggest challenges for developing therapies that are translatable in humans. This phenomenon completely makes sense because ‘you get what you select for’ because the proper selection pressure is key to the success of such high-throughput screening efforts. To improve the translatability of novel capsids, the optimal way in theory would be to perform clinical studies, which is practically infeasible in most cases. Current solutions focus on testing in animal models close to humans, e.g., old world primates, and in multiple models (multiple species, cultured cells, humanized animal models, and organoids). By this means, the risk of low cross-species translatability could be reduced. For instance, a novel liver-tropic capsid, LK03, was discovered through screening in a humanized mouse model with human hepatocytes [93]. Now, LK03 is being tested in two clinical trials for hemophilia A (NCT03003533) and methylmalonic acidemia (NCT04581785). One caveat is that selection in cultured human cells or organoids would help, but there is a significant difference in AAV transduction efficiency in vitro and in vivo. The ultimate way is to identify the receptors responsible for BBB penetrance or cell-specific targeting. Then, we will be able to more reliably predict the performance of novel capsids in human patients. This would also potentially enable the pre-screening of patients based on the expression levels of a given receptor. On the other hand, the efforts to identify receptors for novel capsids have significant theoretical meanings, as AAV transduction is a complicated and yet-to-be-elucidated process that involves multiple steps that can affect transgene expression [89]. Understanding interactions between motifs of AAV capsid proteins.

### 3.2. Cell-Specific Specificity

Currently, the low delivery efficiency necessitates the use of high AAV doses, resulting in high toxicity and cost. Therefore, improved delivery efficiency brought about by next-generation vectors will allow for the usage of lower AAV doses and subsequently reduced safety risks and manufacturing costs. In addition, the specificity of current AAV capsids is mainly at the tissue level, with several tissues being hard-to-reach, e.g., kidney and brain. Therefore, it still remains a challenge to use AAV for treating diseases associated with tissues like the kidney and heart, which impact many patients. As more and more efforts are being made in this field, it is expected that most tissues can be efficiently targeted by next-generation AAV capsids in the future. More importantly, the next step of capsid engineering will be to improve the specificity at the cellular level. Many diseases originating from a certain tissue do not impact all cell types within that tissue. In these cases, it will be ideal to only target those affected cell types, e.g., neurons/astrocytes/microglia cells in the CNS, Schwann cells in the peripheral nervous system (PNS), and hepatocytes/Kupffer cells in the liver. This will further reduce potential off-target risks and free genome space for cargo optimization. One case in point is DRG toxicity from AAV delivery observed in both preclinical and clinical studies [9,94]. A method to address this issue was by including a miR-142 element to minimize transgene expression in DRG [95]. A cell-specific capsid would minimize off-target transgene expression in DRG automatically, rendering the inclusion of miR-142 unnecessary.

### 3.3. Modular Engineering to Improve Multiple Properties

Another challenge for capsid engineering is how to avoid improving one property at the expense of other properties. Some capsid properties are intrinsically coupled with each other for still unknown reasons, and changes made to improve one property may cause a loss of other properties and even basic functionality (e.g., packaging, transcription). It was shown that the initial attempt at fusing an antibody fragment into the AAV capsid to improve specificity led to poor titers [96]. There are three key properties of a capsid: specificity, low immunogenicity, and manufacturability. The optimal solution will be to enable modular engineering that improves multiple properties simultaneously, or at least improves one property while maintaining other properties. It is expected that machine learning can reduce the burden of multi-property optimization through in silico screening.

### 3.4. How to Increase Capacity

Another bottleneck of AAV gene therapy is the relatively small capacity (~4.7 kb), which limits the application where the target therapeutic gene is too large [97,98,99]. One method to overcome this relies on the development of mini-genes that maintain the functions of the therapeutic gene and can be packaged together with regulatory elements in AAV. One representative example would be the discovery of mini-genes for dystrophin [100] and factor VIII [101]. These mini-genes have been tested in clinical trials for hemophilia A (NCT03061201) and Duchenne muscular dystrophy (NCT00428935), respectively. Another approach is the split AAV system, which relies on homologous recombination within the AAV genome [102,103], trans-splicing between transcripts or proteins [104,105], or both [106]. The main drawback of this approach is its low efficiency and the subsequent need for a high dose (potentially >1 × 10^14^ vg/kg), which prohibits further clinical development. Another drawback is the possibility of generating unwanted short sequences, which could be toxic. However, AAV engineering for increased capacity still remains a relatively unexplored field due to the lack of biological understanding of AAV packaging and technical difficulties. Thus, this represents a key future direction for AAV engineering.

## Figures and Tables

**Table 1:** List of approved AAV gene therapy drugs. EU, European Union.

Drug Name	Indication	Sponsor	Serotype	Location	Time
Glybera	Lipoprotein lipase deficiency	UniQure	AAV1	EU	2012
Luxturna	RPE65 mutation-associated retinal dystrophy	Spark Therapeutics	AAV2	USA, EU	2017, 2018
Zolgenmsa	Spinal muscular atrophy	Novartis/Avexis	AAV9	USA, EU	2019, 2020
Upstaza	AADC deficiency	PTC Therapeutics	AAV2	EU	2022
Roctavian	Hemophilia A	BioMarin	AAV5	EU	2022
Etranacogene dezaparvovec	Hemophilia B	UniQure/CSL	AAV5	USA, EU	2022

EU = European Union.

**Table 2:** Comparison of capsid engineering techniques.

Technique	Advantages	Disadvantages
Rational design	-Smaller capsid library size -More efficient workflows for capsid generation -Isolate capsids targeting specific cellular subtype or function	-Requires in-depth knowledge of AAV structural biology -Lesser diversity of capsid variants -Low-throughput discovery yield of novel capsids
Directed evolution	-Larger capsid library sizes -Greater diversity of capsid variants -Doesn’t require knowledge of AAV structural biology -High-throughput increases the discovery yield of novel capsids	-More challenging library design strategies -Unreproducible engineering efforts due to random nature -Little mechanistic insight into improved capsid performance -Few animal models for selection and screening
In-silico design	-Closed-loop engineering using rational design and directed evolution -Identify diverse and functional capsids using previously trained data -Simultaneous optimization of multiple functional parameters	-Time-consuming workflows -Requires high-quality robust training datasets -Issues with availability and cost of computational cloud platforms

**Table 3:** Clinical trials using engineered AAV capsids.

Indication	Start Date	Phase	Trial ID	Biologic Name	Sponsor	Serotype
DMD	2006	Phase 1	NCT00428935	d3990	Nationwide Children’s Hospital	AAV2.5
LHON	2014	Phase 1	NCT02161380	scAAV2-P1ND4v2	University of Miami	AAV2tYF
X-linked Retinoschisis	2015	Phase ½	NCT02416622	BIIB-087	AGTC	AAV2tYF
Hemophilia B	2015	Phase 2	NCT02484092	SPK-9001	Spark Therapeutics	AAV-Spark100
Hemophilia A	2017	Phase 1/2	NCT03003533	SPK-8011	Spark Therapeutics	AAV-LK03
Hemophilia B	2017	Phase 1/2	NCT03369444	FLT180a	University College London	AAVS3
Hemophilia B	2017	Phase 2	NCT03307980	SPK-9001	Spark Therapeutics	AAV-Spark100
Neovascular AMD	2018	Phase 1/2	NCT03748784	ADVM-022	Adverum	AAV2.7m8
RP	2018	Phase 1/2	NCT03326336	GS030	GenSight Biologics	AAV2.7m8
XLRP	2018	Phase 1/2	NCT03316560	AGTC-501	AGTC	AAV2tYF
Diabetic macular edema	2020	Phase 2	NCT04418427	ADVM-022	Adverum	AAV2.7m8
XLRP	2020	Phase 1/2	NCT04517149	4D-125	4DMT	4D-R100
Fabry	2020	Phase 1/2	NCT04519749	4D-310	4DMT	4D-A101
Neovascular AMD	2021	Phase 1	NCT05197270	4D-150	4DMT	4D-R100
Wilson’s disease	2021	Phase 1/2	NCT04537377	VTX-801	Vivet Therapeutics	Anc80
MMA	2021	Phase 1/2	NCT04581785	LB-001	LogicBio	LK03
CF	2022	Phase 1/2	NCT05248230	4D-710	4DMT	4D-A101
Gaucher	2022	Phase 1	NCT05324943	FLT201	Freeline Therapeutics	AAVS3

DMD = Duchenne muscular dystrophy; LHON = Leber hereditary optic neuropathy; AMD = age related macular degeneration; RP = retinitis pigmentosa; XLRP = X-linked retinitis pigmentosa; MMA = methylmalonic acidemia; CF = cystic fibrosis.

## Data Availability

Not applicable.
